# Seeding long-term, sustainable change in teacher preparation programs: the case of PhysTEC

**DOI:** 10.1186/s40594-018-0134-3

**Published:** 2018-10-03

**Authors:** Kathleen T. Foote, Alexis V. Knaub

**Affiliations:** 1 Department of Physics and Astronomy, University of British Columbia, 6224 Agricultural Road, Vancouver, BC V6T 1Z1 Canada; 2 Department of Physics and Astronomy, Biomedical and Physical Sciences, Michigan State University, East Lansing, MI 48824 USA

**Keywords:** Organizational change, Change in higher education, Sustainability of reforms, Secondary teacher preparation, Physics teacher preparation, Dissemination

## Abstract

**Background:**

The continuation of teacher preparation activities after a 3-year Physics Teacher Education Coalition (PhysTEC) grant is used as a case study to examine multi-faceted aspects of sustainable change in higher education. Since teacher preparation is outside typical physics departmental activities, success is highly dependent on finding a department and institution who values this cause. Throughout the history of providing grants, PhysTEC has identified ten components of successful sites that they consider during the selection process. In this paper, we retrospectively analyze characteristics of six comprehensive PhysTEC sites, to see how department histories, values, and activities affect long-term sustainability as sites moved from grant funding to matched institutional funding and beyond.

**Results:**

The most important components required to sustain these programs were (1) institutional commitment—both financial support as well as intellectual and cultural support for potential teachers—(2) champion, a respected change agent at the university who ensures program success through advocacy and support, and (3) activities that enhance not only the production of teachers but also the undergraduate education activities of the department. Of the six PhysTEC sites, three sites were able to institutionalize the majority of PhysTEC activities into departmental routine. These three sites have departmental leadership and administrators who valued and invested in physics teacher preparation. At these sites, PhysTEC symbiotically supported typical departmental activities including increasing majors, improving courses, and involving undergraduates to support teaching. Two sites were sustaining activities at the time of study but attitudes toward teaching as a profession were mixed so continued sustainability is precarious and reliant on external funding. One site discontinued the majority of PhysTEC activities because of a lack of alignment with a different physics teacher initiative on campus.

**Conclusions:**

Because physics teacher preparation is not often prioritized as a part of undergraduate departmental activities, success emerges when departmental and institutional value systems align with this goal. PhysTEC funding is not enough to create this culture; it must exist prior to funding. Sustaining PhysTEC activities is easier when they are seen as enhancing the undergraduate experience as a whole. The PhysTEC grant helped bring physics teacher preparation to the forefront, and a well-respected champion in a leadership position can help set this tone and advance departmental activities accordingly. This study has implications for sustaining reforms of typically undervalued activities in higher education or secondary teacher preparation programs in any discipline.

## Background

Every year, the Department of Education and National Science Foundation (NSF) spend millions of dollars on projects to improve STEM education. While these initiatives aim to create positive and lasting change, the continuation of benefits can be threatened after funding has ceased. Changing departmental demands, competing priorities for faculty members, and administrative and staff turnover can threaten reforms, especially when knowledge and expertise are lost along the way (Coburn 2003; McLaughlin and Mitra 2001). Efforts heavily driven by external support can be temporary solutions that regress when support is removed (Chasteen et al. 2015). Change is notoriously difficult in realms of public service, such as education, health, and social welfare because improvements may not directly lead to commercial gain (Elwyn et al. 2007). In these industries, it may be especially challenging to encourage people to adopt innovative, research-based practices if they lack financial or industrial pressures to change.

In past studies, we found that hard-to-reverse structural changes, such as replacing lecture halls with studio classrooms like SCALE-UP, are one way to counter this trend (Foote et al. 2016; Knaub et al. 2016). However, most reforms do not involve structural changes. As funding for higher education becomes increasingly squeezed within institutions, understanding how reforms, even those outside typical departmental priorities, can have a sustained impact which is critical to creating meaningful, lasting change. Sustained change often requires more than a change in practice; it involves aligning the culture with the change, ensuring values, beliefs, and rituals help institutionalize the reform amidst changing administrators, budgets, and staff expertise (Schein 1985). In this study, we found that culture and history are a resilient part of a departmental identity. If departments do not value physics teaching as a profession, the funding is unlikely to change faculty and student attitudes. This value system will not emerge from the funding; it must already exist.

This study seeks to understand how to align desired changes with the local site so change can be executed strategically and efforts can succeed long term. To use an agricultural metaphor, external change agents can widely scatter information about initiatives relying on chance for those efforts to succeed; or one could actively seed efforts in fertile soil where growth and further propagation is likely (King 2003; Gannaway et al. 2013). This process involves two main stages: first, funding agencies and change agents need to know how to identify sites that are open to change and have histories, cultures, and structures that are aligned with the intended change. Secondly, during the grant period, change agents should focus on capacity building in strategic areas that are linked with long-term sustainability that the programmatic efforts continue after the grant funded period.

While organizational change models are well-established in business settings (Real and Poole 2005), less work has been done to articulate and refine models for change in higher education (Chasteen et al. 2011; Henderson et al. 2011). Many of these emerging models lack a unified theoretical foundation and focus primarily on changing practice (e.g., having instructors use active learning techniques) in teaching and learning. Getting university departments to invest long-term in producing physics teachers requires much more than a temporary change in practice because teacher preparation is outside typical physics departmental duties in higher education. We argue that to sustain any change in practice in a resilient way, culture and value systems need to align with the change. To avoid failure, sites should analyze alignment before attempting reform.

We chose the Physics Teacher Education Coalition (PhysTEC) as a case study because it helps make the role of value systems in change efforts explicit, because creating physics teachers is not a common priority in physics departments. Since 2001, PhysTEC has been using NSF funding to help universities develop and transform physics teacher programs according to national models. They have modified their application and funding policies to explicitly focus on sustainability (Scherr et al. 2014) and now require applicants to submit a sustainability plan. For example, the first sites were funded for 5 years but current comprehensive grants will fund 3 years under the condition of 3 years of matched institutional funding. While an in-depth examination of sustainability of early sites has already been completed (Scherr et al. 2014), we study the sustainability of sites under this new matched funding model, to see how results compare to the data collected in 2012. We situate our results within change models, so funding agencies and change agents can structure their application, selection, and implementation guidelines in a way that increases the probability of sustainable outcomes. We hope to generalize some of what works well for PhysTEC grants by situating results within the change literature to guide efforts of funding agencies, change agents, and leaders in higher education more broadly.

We hypothesized that teacher preparation programs would be difficult to sustain because producing secondary teachers is outside typical university physics department missions. “Physics teacher preparation at colleges and universities generally has an ‘orphan’ status, claimed or valued by almost no one, except as a low-priority side-line activity” (Meltzer et al. 2012). The small number of graduating physics teachers can make the dedication of staff, courses, and resources difficult to justify if secondary education is not valued. We hope this paper makes an implicit criteria for change (aligned values) explicit and provides practical exams of how departmental value systems interact with change efforts.

While many physics faculty do not see producing physics teachers as a responsibility of university departments, the severe lack of qualified secondary teachers creates a dangerous cycle that has significant implications for university departments. As a result of poor (or no) physics instruction in high school, few high school students are inspired to pursue further study of physics. This limits the potential for physics majors, putting physics degree programs at risk of elimination due to low enrollment. The jobs of physics faculty, and the resources allocated to the department, are affected by students’ experiences in secondary school. Maintaining the status quo has negative implications for physics as a discipline and the United States’ ability to maintain scientific and technological competitiveness (Meltzer et al. 2012).

For changes to be institutionalized, Gannaway et al. (2013) proposes sites think about creating climate for change, engaging key parties, and transferring grant activities to sustainable funding sources. Strategically identifying sites where change efforts are aligned with existing departmental culture and structures is the first step toward investing in meaningful change. While PhysTEC does have “key components” that they review in applications for funding, this study will shed insight on which of those components were critical for the sites studied here.

### Climate

Explicitly assessing the climate of readiness for change frees a project from being dependent on serendipity and luck. Before embarking on a project, one should consider of the capacity of people and systems to change, or at least able to be convinced that change is worthwhile. Assessment of the climate’s readiness for change begins with understanding the culture and organizational structures at the sites where change is anticipated (Gannaway et al. 2013).

As institutions with long-standing missions, universities tend to value history, traditions, and often institutional processes reinforce existing practices. Universities have a unique leadership structure which can be quite hierarchical at the upper levels but is rather decentralized at the departmental level with individual faculty having a high degree of autonomy in certain areas. Despite this unique leadership structure, universities share some common features with other organizations. The broad literature on organizational change provides some ideas on how to best change university culture. In this paper, we decided not to restrict our interpretation by using one change framework. Instead, we draw from a variety of literature with applicability to higher education.

Organizations have a variety of features that enhance their ability to embrace and assimilate new ideas (Pettigrew et al. 1994). Components of receptive context include strong leadership (Cooper et al. 1998), clear strategic vision, positive administrative relations, visionary people in pivotal positions, a climate conducive to experimentation, and ability to continually assess progress (Anderson and West 1998; Dopson et al. 2002; Newton et al. 2003; Nystrom et al. 2002; Pettigrew et al. 1994; Van de Ven et al. 1999). Strong leadership is often at the root of developing a culture where members can break out of the convergent thinking, take risks, and try new things (Van de Ven et al. 1999).

Innovations that are compatible with the intended adopters’ values, norms, and perceived needs are more readily adopted (Aubert and Hamel 2001; Denis et al. 2002; Ferlie et al. 2001; Foy et al. 2002; Rogers 2010). Thus, the climate for change also depends on the characteristics of the specific proposed project process, and outcome. When deciding whether change is worth the effort, people within the institution weigh the intended impacts and perceived benefits, deciding whether it addresses a perceived need.

### Engagement

Engagement includes the involvement of invested key stakeholders, champions, and change enablers (Gannaway et al. 2013). Kotter recommends that an organization must assemble a group with power, energy, and influence to lead the change. The leadership team should include members with (1) positional power so opponents cannot block progress, (2) necessary expertise to make informed decisions, (3) sufficient credibility for the organization to enact decisions, and (4) leadership to inspire progress (Kotter 1996). Collective leadership improves the chances of arriving at more complex, complete solutions and eventually achieving a broader impact (Kezar 2013). It also promotes widespread buy-in in the face of administrative and staff turnover.

PhysTEC defines a champion as a change agent at the university who ensures program success by stepping up as an advocate when support is needed (Meltzer et al. 2012). The champion is usually a faculty member who creates, funds, staffs, and institutionalizes physics teacher education at their institution. PhysTEC also describes the need for institutional support, which encompasses a wide variety of people from physics and education faculty, as well as chairs, deans, and upper-level university administration. Deans and upper-level administrators often contribute financial support and dictate institutional priorities, while much of the student-facing communication happens at the departmental level.

The department translates administrative visions and priorities into how it affects the daily functioning of faculty. Similarly, departmental norms and rules shape the ways in which faculty members interpret and enact their roles and responsibilities in relation to the larger university (Seymour 2002; Wieman et al. 2010). The early and widespread involvement of people at all levels enhances the success of implementation and eventual institutionalization (Kotter 1996).

### Sustainable transfer

We define sustainability using three operational indicators: (1) maintenance of a program’s intended benefits (in this case, production of physics teachers), (2) institutionalization of the program within the department, and (3) capacity building in the local setting (Rabin et al. 2008; Shediac-Rizkallah and Bone 1998). Capacity building is any activity that builds durable resources and enables the organization to continue the delivery of the change after external funding ends. This can involve training of internal staff, identification of alternative resources, and building internal assets (Shediac-Rizkallah and Bone 1998; Pluye et al. 2004). While we look to see if particular activities are sustained, to understand the importance of these components, ultimately, producing physics teachers is the goal of PhysTEC and the basic criteria for sustainability.

Jacobs (2002) defines institutionalization as change that has relative endurance and staying power over a length of time or that “has become part of the ongoing, everyday activities of the organization”. Practice becomes institutionalized when it is routine, widespread, legitimized, expected, supported, permanent, and resilient (Kramer 2000). Three stages that determine the extent of institutionalization are (1) passage—a single event that involves a significant change in the organization’s structure or procedures such as transition from temporary to permanent funding, (2) cycle or routine—repetitive reinforcement of the change by including it into organizational procedures and behaviors, such as the annual budget and evaluation criteria—and (3) niche saturation—the extent to which the change is integrated throughout organization (Shediac-Rizkallah and Bone 1998; Pluye et al. 2004; Johnson et al. 2004).

## Participants and data collection

### PhysTEC

PhysTEC began in 2001 to combat the severe shortage of qualified high school physics teachers as a partnership between the American Physical Society and American Association of Physics Teacher with funding from the NSF. Since inception, PhysTEC has directly supported over 40 institutions to build model programs to educate future physics teachers, and PhysTEC-supported sites have collectively more than doubled the number of graduates per year from their physics teacher preparation programs (Meltzer et al. 2012). Unlike other teacher preparation programs, the PhysTEC project targets physics departments when creating a supportive climate toward teaching and encouraging embedded activities.

Some other teacher education efforts overlap with PhysTEC efforts, as will be discussed in this study. For example, UTeach is a STEM education initiative out of University of Texas at Austin. This initiative started as a collaboration between the College of Natural Sciences, the College of Education where they brought experienced “Master teachers” (secondary school teachers) to campus to lead introductory courses and coordinate on-going field-based experiences (Petrosino and Dickinson 2003). Since starting in Texas, 46 universities in 22 states participate in UTeach including 2 of the sites in this study (UTeach 2018).

The Robert Noyce Teacher Scholarship Program was first authorized by the NSF in 2002 to address the shortage of STEM teachers and professionals (AAAS 2018). This program provides funding to tertiary institutions to provide scholarships, stipends, and programmatic support to recruit and prepare STEM majors to be physics teachers. Students who receive scholarships are expected to teach for 2 years in a high needs school district. All of the sites in this study had Noyce funding at some point.

This study focuses on comprehensive sites that received grant funding for 3 years under the condition of matched institutional funding for an additional 3 years. PhysTEC sites benefit from not starting from scratch since the PhysTEC only awards grants to applicants who already demonstrate certain features of successful teacher preparation programs, including a faculty champion, institutional commitment (in terms of matched funding), and collaboration with education departments. This study intends to shed insight on which of these pre-conditions might be more important than others in creating sustainable outcomes.

Under the grant, comprehensive PhysTEC sites are encouraged to adopt additional components including recruiting pre-service physics teachers, offering early teaching experiences, advising and mentoring teacher candidates, and connecting graduates to a network of local teachers. Learning assistants, talented undergraduates who help support student learning in undergraduate courses, are often used as a way to provide early teaching experiences, while enhancing the quality of undergraduate teaching (Otero et al. 2010). Often these students participate in supplemental courses in physics teaching methods, where they learn pedagogical content knowledge.

PhysTEC has changed their list of components over the years to prioritize activities that do not typically exist at universities. For example, they used to include “course transformation” because that was linked to demonstrating high-quality teaching practices that could attract more majors, which expanded the number of potential teachers. Now, the listed components have more direct impacts on producing physics teachers. While each site adopts key components as a part of the grant, sites are encouraged to implement them according to their local context, taking advantage of institution-specific resources and expertise (Scherr et al. 2014; Chasteen et al. 2018).

Recent requests for proposals for new sites explicitly ask for sustainability plans, and all awardees featured here (except CSU-LB) committed upfront to sustain their programs for three or more years beyond the PhysTEC award period. Expectations for sustainability were much less clear at the beginning of the project, which included several sites in the first sustainability paper (Scherr et al. 2014). We wanted to see whether explicitly mandating sustainability plans and matched funding (for institutional commitment) lead to significantly different sustainability outcomes compared to when data was collected in 2012, where some awards were given prior to this requirement.

In the first sustainability report, with data collected in 2012, Scherr et al. (2014) found that seven out of the eight sites sustained or expanded production of physics teachers. All seven of these sites had both a faculty champion and institutional motivation and commitment, by design of the selection criteria. Institutional motivation included examples such as fulfilling the institutional mission, serving regional needs, improving the local reputation of the institution, supporting strong members of the faculty, and national recognition. They found that every site that sustained an increase in production of physics teachers continued recruitment efforts, “providing evidence that recruitment is necessary for sustaining increases in teacher production” (Scherr et al. 2014, p. 8). Scherr et al. (2014) found that sustained activities tended to be those that fit in well with normal departmental interests and responsibilities (e.g., LA program to support undergraduate teaching), while other activities (e.g., secondary outreach) tended to be reduced or eliminated.

The one site that did not sustain activities had little support for physics teacher preparation prior the grant. Since the grant, the university’s teacher certification program was eliminated and the colleague in the department of education retired when the department closed. The grant was used to fund a marketing campaign, course transformation, and undergraduate teaching experiences within the university. The course transformation, teaching experiences, and recruiting has continued, but students would need to receive a certificate through another avenue so the number of teachers has dropped, as well as mentoring opportunities.

Our study seeks to revisit the long-term sustainability of sites after changes were made to require matched funding. We will generalize and situate findings within the larger change literature.

### The sites

We investigated the six PhysTEC comprehensive sites who had completed funding by 2014 and who were not featured in the 2014 sustainability report (Scherr et al. 2014). The year 2014 was chosen so that the post-funding period between 2014 and 2017 could be studied to investigate long-term sustainability. All six sites that met these criteria participated. These sites are described in Table 1 using their Carnegie Classification. Five of the six sites are at least 3 years post-grant funding and have thus faced two funding transitions (from grant to institutional funding, then beyond required matched funding). While the sixth site (VTech) is still technically within the institutional funding period, they took steps to institutionalize changes with sustainable funding sources which justified their inclusion in this study. The sites are a mix of research universities and masters colleges, some of which historically specialized in producing teachers (Towson and CSULB).

**Table 1:** Carnegie classification of sites and approximate number of faculty in physics department

Name	Code	Fraction of undergraduates at institution	Masters (M) college/doctoral (D) program	Number of faculty in Physics Department
Boston University	BU	Majority	D- highest research activity	45
California State University- Long Beach	CSULB	High	M	22
Middle Tennessee State University	MidTenn	Very high	D- moderate research activity	15
Towson University	Towson	Very high	M	27 (includes astronomy and geoscience)
University of Minnesota	UMN	Majority	D- highest research activity	65 (includes astronomy)
Virginia Polytechnic Institute and State University	VTech	High	D- highest research activity	36

As seen in Table 1, the sites are a diverse group of research institutions and masters colleges of varying sizes. For example, CSULB and Towson have no PhD program which means they cannot recruit graduate students to serve as teaching assistants in reformed courses, laboratories, and tutorials. Conversely, the high research focus of UMN, VTech, and BU may have implications for how much time research faculty can devote to teaching and how teaching is valued and rewarded. The high level of variability makes quantitative comparisons difficult because the circumstances, initial conditions, and contextual differences of site vary dramatically. That is not the purpose of this paper. Instead, we aim to provide qualitative descriptions of how each site navigates the change process in light of its unique strengths and challenges, and can leverage strategic aspects of its identity, culture, and history to create permanent transformation. Each institution will be described in more detail in subsequent sections of the paper.

### Data collection

With the help of the PhysTEC leadership, we reached out to at least two key informants (including the champion in the physics department) at each institution, conducting a total of eleven interviews. The second person we talked to was often a champion in education (at VTech, CSULB, UMN, and BU). At Towson, we talked to two science educators in the physics department. We had not originally planned to interview TIRs because we expected them to execute and implement the vision developed by champions, faculty members, and administrators. However, in the case of two sites (BU and VTech), the TIRs ended up staying more than the year recommended by PhysTEC and took a leadership role in ensuring the continued existence of their position as well as supporting other PhysTEC efforts. In these cases, when the TIR became an active advocate and creator of change, we felt their perspectives were relevant to our research questions.

The hour-long telephone interviews highlighted key factors that our interviewees perceived as important. Interviewees were asked open-ended questions about the context (e.g., events that motivated applying for PhysTEC, allies in education and higher administration), implementation (e.g., grant activities, challenges, successes), and transfer (e.g., grant components that had been maintained during transitions, future outlook). The interviewer used follow-up questions to obtain specific details about the interviewee’s experiences. The common interview protocol was adjusted to the interviewee’s role, e.g., slightly different for teachers in residence (TIR) compared to the department heads. Interviews were transcribed for analysis.

Interviews were supplemented and cross checked with a variety of documentation most of which was submitted to PhysTEC throughout the grant period, which involved the perspectives of external and internal stakeholders. While the interviews are short, this documentation provides incredibly valuable information about details of how the grant unfolded over time. Documentation included annual, midyear, and data reports, as well as notes from external site visits conducted by the APS PhysTEC staff. The documentation and interviews were triangulated to explore the history, evolution, stresses, opportunities, motivations, and constraints on physics teacher preparation at each site.

### Data analysis

Individual interviews were transcribed, reviewed, and summarized with structured note-taking. Interviews at the same institution were compared and crosschecked with PhysTEC documentation (including annual and midyear reports) and university websites to ensure consistency. Both researchers separately summarized the interviews and documented components of the change process and aspects related to sustainability. We compared our notes, resolved any discrepancies, and typically combined responses in a more complete summary. Finally, interviews were compared across institutions to highlight thematic findings.

The PhysTEC team and featured sites checked these findings for accuracy. Furthermore, the documentation submitted to PhysTEC over the grant period was validated by its members before being viewed by us. We sent the draft of this manuscript to each site for further verification.

## Results

In “PhysTEC” section, we provide a brief synopsis of each site that describes their relevant history leading up to the grant, how they used PhysTEC funding, and whether they were able to maintain teacher production, institutionalize activities, and further build capacity to internalize changes after funding ended. In “The sites” section, Table 2 summarizes these results.

**Table 2:**
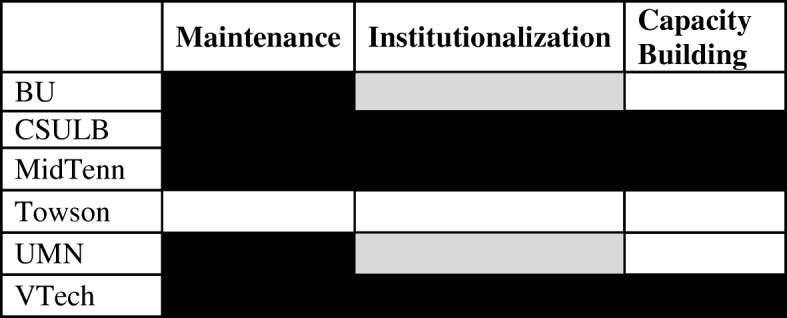
Summary table of sustainability at sites, with “black” indicating strong sustainability, “gray” indicating tentative sustainability (for example, dependent on another source of external funding). “Maintenance” refers to continued production of physics teachers. “Institutionalization” refers to continuation of PhysTEC activities, such as the TIR, recruitment, pedagogy courses, etc. “Capacity building” indicates that costs and staffing has been internalized by the university

### Synopsis of each site’s grant activities and sustainability outcomes

#### Boston University

Boston University (BU) is a private, urban, R1 institution and was the first PhysTEC site in New England. The principal investigators (PIs) on the PhysTEC grant were a Master Lecturer in Physics and a Clinical Associate Professor in Education. Prior to the grant, the PIs worked together to provide professional development to teachers at local schools when the district switched to a Physics First model where physics is the first high school science course. Because of this project with in-service teachers, the PIs started attending PhysTEC meetings, where they met their future TIR and where they were inspired to apply for the grant and start an LA program.

##### Successes

The TIR has been a critical part of BU’s PhysTEC efforts, actively recruiting students and career changers through listservs for physics teaching. He started the Boston University Physics Teachers Network that meets on campus five times annually with high participation (200 teachers on the mailing list) in the local community. The TIR has been involved on additional projects to secure external funding, including creating an online and in-person AP physics course for local schools and Noyce scholarship work.

BU’s PhysTEC efforts included expanding their LA program, including using LAs to support studio teaching in first year courses. After their PhysTEC funding ended, BU’s LA program has continued to flourish, with funding from the institution to support over 140 undergraduates throughout the institution per year. The LA program is essentially institutionalized, but the undergraduates view it more as a way to prepare them to teach during graduate school, instead of inspiring them to teach high school. The TIR helped the department navigate the transition to studio teaching, and now that is also institutionalized.

##### Challenges

One of BU’s PhysTEC aspirations was to establish a teaching track for undergraduates (in addition to the existing pathway of a Masters of Arts in Teaching), which passed the faculty vote in physics but was ultimately voted down by the School of Education. The PI in physics identifies the failed pathway as his biggest regret, but does not believe he could have done anything to change the outcome.

While the TIR believes faculty in the physics department are supportive of his efforts, getting widespread participation in secondary outreach efforts is difficult. BU continues to struggle to recruit undergraduates who fail to see teaching as a prestigious and lucrative career, considering the high tuition for their degree. The co-PI in education developed a two-credit course modeled after UTeach to give undergraduates an early teaching experience but enrollments were low.

When we first interviewed representatives from BU, the continuity of the TIR, who led the majority of PhysTEC efforts, was contingent on finding an external funding source beyond the Noyce scholarship and digital learning grant related to developing the online AP physics course. Since then, the faculty champion worked with the TIR to secure NSF funding to support his continued employment. The PIs have asked for departmental funds to sustain on-campus networking events with their extensive physics teacher network.

##### Current status

Before PhysTEC, BU only graduated one physics teacher per year. At one point during the grant, they were producing more than five per year. Now, BU graduates 3–4 teachers per year. We characterize BU as *maintaining teacher production* but only *moderately institutionalized* because funding to keep the TIR, who leads recruitment efforts, is dependent on external funding. The undergraduates at the university and many faculty within the department do not view teaching as a prestigious career, the department is not particularly eager to embrace secondary teacher preparation as part of its mission.

#### California State University Long Beach

California State University Long Beach is a public, urban, masters’ institution with an excellent reputation for teacher education historically. California State University Long Beach (CSULB) produces about 400 elementary school teachers a year and has more applicants than they can accept. The PI in physics became interested in the project because she was disappointed with the quality of her son’s science education and thought it might help remedy the low number of physics majors (around ten per year). She joined forces with the Chair of Science Education and an undergraduate advisor who was a popular instructor and advocate for instructional technology to apply for PhysTEC funding.

##### Successes

Because the Chair in Science Education had extensive experience with educational grants, she recommended limiting PhysTEC activities to high impact, sustainable activities with low ongoing costs. Because of this, almost all activities were sustained. The Chair of Science Education, who had the most experience with teacher preparation projects explains,

We picked… things that I think had the biggest impact for our local teacher community and for our prospective teachers and that opportunity to get the networking… For our future teachers can be in the same room with the experienced teachers on a somewhat regular basis, they’re starting to build their network. They’ve got classrooms they can go to and they have to do early fieldwork experiences. Now they know five or six or ten physics teachers in the area.

Specifically, they started a biannual open house—attended by the Provost, monthly demonstration-sharing lunches with local teachers and created a monthly newsletter in collaboration with the Southern California American Association of Physics Teachers. Initially, it took time and effort to seek out secondary teachers and create interest in these activities. However, once the networks were established, the logistics for Open House events were arranged, the format of the demonstration sharing lunches were determined, and it required minimal resources to continue them, costs that were easily picked up through some Sumner Foundation funding once the grant ended.

Since recruiting a qualified full-time TIR was difficult, they chose to have a part-time TIR and did not use the full comprehensive funding of $100,000/year. CSULB was the only site in this sample that was awarded the grant before PhysTEC introduced the matched institutional funding requirement. The part-time TIR started two new courses: a LA pedagogy course and a physics pedagogical content knowledge course. While PhysTEC usually recommends full-time TIRs, CSULB chose to accept less grant money for a part-time position, which also made it easier to recruit teachers. The champion from CSULB also believed PhysTEC award helped distinguish physics in the faculty of science and helped secure support from administration. For example, when the grant ended, the position continued with Math Science Teacher Initiative funds from the Chancellor’s office.

CSULB was able to *sustain all the major components with slight modifications*. Specifically, they reduced the frequency of the Open House and newsletter. Looking back, the Physics Chair and PhysTEC champion reflects that their highly focused application made it possible to accomplish activities and continue to do those things after PhysTEC ended. For CSULB, setting limited, high-impact but realistic targets resulted in extremely high sustainability.

##### Challenges

The hardest part of maintaining PhysTEC efforts is funding for 20 LAs salaries per semester. With encouragement from PhysTEC, CSULB took data to show that students connect strongly with LAs, pass rates went up, and attitudes toward science improved. This data has helped secure university funding for now, but the program may be at risk for being scaled down.

##### Current status

Overall, the PhysTEC champion at CSULB—who was Chair until a year ago—believes the department supports all main components and believes it “wouldn’t come down easily” especially because it has led to an increased number of physics majors.

CSULB has been able to maintain the programmatic efforts started during their PhysTEC funding period, graduating around five physics teachers per year, significantly more than before the grant. They sustain their part-time TIR through teaching responsibilities with the LA and physics pedagogical content knowledge courses. We characterize them as institutionalizing changes and capacity building with PhysTEC through on going recruiting, outreach to local schools, and continued employment of the TIR through LA and physics pedagogy courses.

#### Middle Tennessee State University

Middle Tennessee State University (MidTenn) is a public, suburban institution with moderate research activity. The economic recession and impending budget cuts threatened the existence of MidTenn’s physics department. Introductory courses had high drop, failure, and withdrawal (DFW) rates (around 55%), physics majors were few, and they had not graduated a physics teacher in over 20 years. Within the physics department, there was an obvious need and urgency for change (Kotter and Darius 1997). The PI was a faculty member in physics with an interest in education who had been going to PhysTEC conferences for 4 years before receiving a personalized invitation to apply.

The threat of department elimination successfully led to course improvements preceding PhysTEC. The physics department reformed their introductory, algebra-based classes using a studio format. Transforming the algebra-based courses into a studio format fixed the pressing issue of DFW rates of over 50%. The initial successes and eagerness for recruiting more majors caused them to apply for PhysTEC funding, which inspired further transformation in calculus-based courses with Matter and Interactions (Chabay and Sherwood 2015) curriculum and LAs, which caused further increases in the number of majors. These course reforms helped lower their DFW rates from ~ 55 to 15%.

The university decided to distinguish itself as valuable to the state of Tennessee by rebranding itself as a major source of STEM teachers so the new institutional identity aligned with the goals of the PhysTEC grant. In 2010, the institution committed $2.5 million dollars to becoming one of UTeach sites in the state (PhysTEC 2011), concurrent with the start of the PhysTEC grant and a Noyce Scholarship program for prospective physics and math teachers.

PhysTEC complemented the department’s efforts to recruit more majors in general, by providing a clear potential career path that complemented the university’s effort at rebranding itself as a leader in science education. Thus, the president, provost, and dean all highly supported PhysTEC through verbal encouragement and financial support. MidTenn submitted a PhysTEC application around the same time as establishing a doctoral program in science education and hiring a Physics Education Researcher which helped build capacity because now the departmental had its own expert in pedagogical content knowledge.

##### Successes

The physics champion leveraged both a state-level interest in producing more physics teachers in Tennessee and a departmental-level interest in increasing the number of physics majors. PhysTEC coincided with the university adopting UTeach and securing Noyce scholarships, a combination that worked well together. There was an advisory group for STEM education that included the PI for PhysTEC, the dean of science, vice president for teacher licensure, chair of education, and UTeach leadership. Because the university received UTeach funding, PhysTEC negotiated a reduced $60,000/year award with MidTenn physics. This did not include funds for a TIR, since they had access to a Master Teacher (funded by UTeach) who could fulfill that role. MidTenn’s PhysTEC efforts involved course reform, adding a departmental concentration in physics teaching, marketing materials, LA program, and a program where high school students visit the campus.

##### Challenges

The TIR was deemed a failure because the UTeach responsibilities occupied most of their time and their location on the UTeach part of campus meant they were not a physical presence for physics students. The full teaching degree requires a whole year of student teaching before you can qualify for licensure, which makes it a lengthy process for students. Many students jump into teaching and take classes at night, leading to an underestimate of the number of graduated physics teachers.

##### Current status

Higher administration continues to be supportive especially since the reformed classes have contributed to physics having the lowest drop, failure, and withdrawal rates within the faculty of science. The president of the university does not know much about the drop, failure, and withdrawal rates but noticed the PhysTEC site visits and acknowledged physics as a “program of distinction for undergraduates”.

MidTenn maintained many of these activities including the course transformation and LA program with university funds. The marketing aspect was maintained because once the materials and strategy were developed, the resources remained applicable. The PhysTEC PI is currently the department chair. He spends time inviting high school teachers to campus, in the hopes they will recommend the program to their students. While he believes most PhysTEC activities would continue, he does not know who else in the department would spend time and energy on recruiting.

We characterized MidTenn as *maintaining teacher production* (2–3 per year), *institutionalization of LA program and course reforms*, and *moderate capacity building* since PhysTEC activities have continued through departmental and UTeach activities.

#### Towson University (Towson)

Towson University is a public, suburban, masters’ institution. The Chancellor of the University of Maryland wanted to triple the numbers of science teachers. One of the PIs was one of the first science educators embedded within the physics department, hired 11 years of secondary education experience. This educator partnered with an elementary science education colleague in the department who had started an LA program years prior. The department chair had been active in the AAPT, taught using innovative strategies, and supported PhysTEC efforts as a way to increase physics majors. Their PhysTEC efforts included hiring a TIR, marketing materials, and increasing student recruitment.

##### Successes

While it was difficult to recruit a TIR, they eventually found a veteran teacher who stayed for 2 years, was respected by and connected to local teachers, and extensively recruited potentially secondary education majors. The TIR was respected within the physics department so physics faculty started sending students to talk to him about a future in physics teaching. The TIR offered professional development around Physics with Inquiry, a pedagogical innovation, and started a newsletter with local teachers.

##### Challenges

While they were successful in increasing the number of physics graduates prepared to teach, PhysTEC efforts have been challenging after grant funding ended. The university adopted UTeach (Brainard 2007) as a top-down decision from the provost, even though the deans realized that UTeach’s rigid structure may not work well with the science education expertise located in the physics department. UTeach was largely based in the School of Education and aimed to increase STEM teachers in general (as opposed to physics teachers specifically) with a more rigid structure, where UTeach Masters Teachers would teach courses that the science educators had taught in the past. Because PhysTEC efforts were within the physics department, misalignment between program aims and communication challenges impeded synergy between these two efforts. According to our interviewees (we did not interview representatives from UTeach), the two efforts struggled to find common ground.

##### Current status

When PhysTEC ended, supposedly physics would have access to a Master Teacher as part of the sustainability plan. However, UTeach at Towson lacked a Master Teacher with a physics emphasis, so all the recruiting and marketing activities were essentially discontinued.

Their LA program, which existed prior to the PhysTEC funding, was sustained through funding from the Dean. However, without a TIR to talk to LAs about high school teaching, the interviewees comment that the LAs do not tend to become secondary education majors.

We characterized Towson as *not maintaining production* of physics teachers because numbers have dropped to pre-PhysTEC numbers. We do not consider PhysTEC activities institutionalized, since without a TIR, LAs are not associating teaching experiences with teaching high school. The TIRs play an important role as a physics teacher ambassador, a positive role model for teaching who can explicitly encourage students to pursue it as a future career (Chasteen et al. 2018).

#### University of Minnesota

University of Minnesota, Twin Cities is a public, urban, R1 institution. The PI is an astrophysics professor with interest in physics outreach, the learning assistant program, and improving physics education. The physics department has a history of outreach, and their physics demo road show created a historic connection to local schools, which proved to be valuable in recruiting TIRs. The PhysTEC team involved faculty from physics and astronomy, post-secondary teaching and learning, the college of education, human development, as well as local schools. Producing teachers was not a need that was echoed at the institutional level but they did get university funding for the LA program.

##### Successes

University of Minnesota (UMN) used the PhysTEC grant to fund a TIR, who focused a large fraction of their efforts on the LA program. The TIR recruited and hired LAs, teaches a science pedagogy course for LAs, and successfully helped get course credits from the LA pedagogy course to count toward the Master’s degree in teaching. Data was useful to advocate to the dean to support the LA program. UMN also developed a new program “DirecTrack to Teaching” which allows undergraduates to start taking courses to lead to certification.

##### Challenges

While students enjoy being LAs, it can be difficult to find professors who want LAs in their courses and many LAs are not interested in high school physics teaching.

Additionally, faculty attitudes toward teaching are variable and there is a perception that “students do not attend UMN to become teachers”, as one interviewee stated. This attitude is shared by some faculty and administrators at the institution, including the director of undergraduate studies who had some reservations about recommending high school teaching as a career.

The challenges were succinctly described in one of the final reports: “We are philosophically committed to continuing support of the teachers prepared in our program. However, our resources are currently stretched very thin, both in terms of funding and time.” The PI was writing grants in collaboration with other departments in hopes of getting funding to sustain the LA program and the TIR position.

##### Current status

When we interviewed the PI, the TIR position had been maintained with department funds by paying them to support the LA program. However, funding was extremely uncertain and it remains unclear whether efforts would continue without the PI. The PI is passionate about science education and outreach, but the departmental culture does not widely embrace these as a primary part of its mission.

Thus, we characterized this as maintaining production of teachers and unstable institutionalization because PhysTEC activities and the TIR employment is contingent on continuing to find funding sources.

#### Virginia Polytechnic Institute and State University

Virginia Polytechnic Institute and State University (VTech) is a public, rural, land grant, R1 institution. At VTech, several senior faculty members in leadership positions cared about high school teaching and it aligned with institutional priorities. Around the time of application, the VTech president signed a letter to former President Obama alongside other land grant universities promising to address the nationwide shortage of STEM teachers. The Dean of College of Science, and a former chair of the physics department, started the Physics Outreach Team in the mid-1990s. This team continued as an established part of departmental activities, creating a precedent of valuing outreach. The Department Chair applied for PhysTEC funding with a program leader for Science Education. When this Department Chair left the university to pursue a leadership position elsewhere during the grant, they passed PhysTEC responsibilities to another senior physics faculty member. The new PI in physics had a long friendship with the colleague in education who had fostered his interest in teacher preparation so there was a strong collaboration between the physics and education departments. Although their PhysTEC team lost a leading member, there was no significant harm done because of the collective leadership.

##### Successes

Even before PhysTEC, the collaboration between departments helped support students transition from undergraduate physics to graduate teacher licensure program by funding a Masters in Teaching through a Teaching Assistantship. There were two champions within the physics department so efforts continued seamlessly even when the principal applicant moved to another institution.

The PIs on the grant credit much of their success to the TIR, who was a VTech alumna who had taught for a few years prior to returning to VTech as the TIR. Before PhysTEC funding ended, she became a full-time instructor in the department in introductory and outreach courses. Between outreach and LA opportunities, students are exposed to a wide variety of early teaching experiences available to any physics student. The TIR continues to be a highly visible resource for any students contemplating a career in high school physics teaching. The university switched to studio teaching, and the TIR supported the transition. Administrative support simplified financial transitions and secured the TIR’s continued employment as a departmental instructor.

##### Challenges

Since VTech has a rural location, it is difficult for teachers to visit the university for Teacher Advisory Group meetings. Now that the TIR has more instructional duties, they have less time to visit local schools. It was also slightly difficult to recruit LAs because of busy schedules.

##### Current status

We categorized VTech as *maintaining teacher production* because it continues to meet/exceed the rate produced during the grant. VTech *has institutionalized PhysTEC efforts* and *built capacity* by internalizing costs, hiring the TIR as a full-time instructor, and supporting potential teachers through Teaching Assistantships as they earn their Master’s in teaching.

### Summary chart of major grant components and sustained activities

In Table 3, grant activities are often modified to some degree over time but since it is difficult to quantify the “degree of sustainability,” we chose to divide the activities into “sustained” or “high risk/ not sustained” based on whether the primary function of the component was still being fulfilled at the time of study.

**Table 3:**
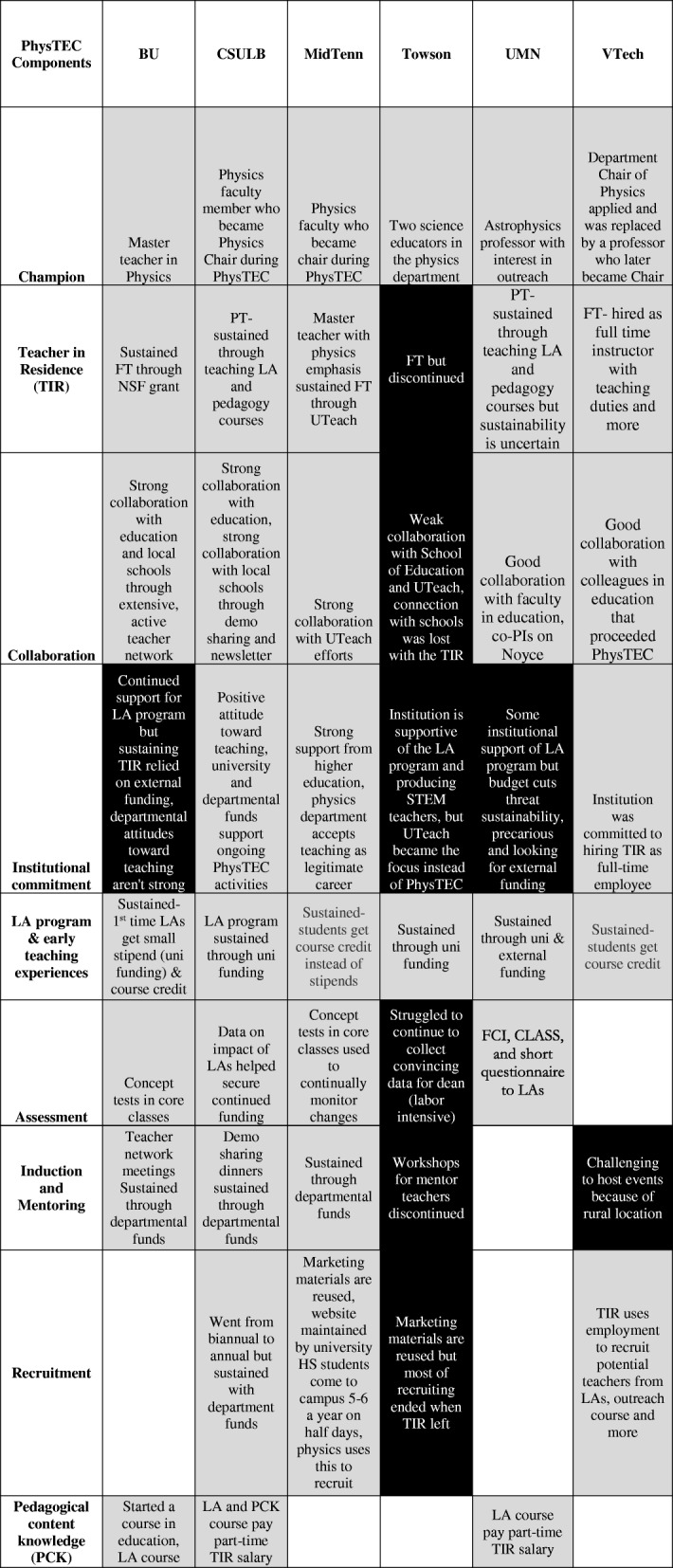
Sustainability of major grant components. Gray indicates “sustained,” black indicates “high risk/ discontinued.” PT and FT are part-time and full-time respectively. Blank cells indicate that component was not a significant part of grant activities at that institution

## Discussion

The following section highlights key themes regarding how sites did (or did not) create fertile soil for sustainable change. As mentioned by King (2003), fertile soil involves both (1) favorable pre-conditions and (2) capacity building during the grant period in strategic areas.

### Connecting PhysTEC to essential departmental duties

Our study reinforced Scherr et al.’s (2014) conclusion that sustained activities tended to be those that fit in well with normal departmental interests and responsibilities (e.g., LA program to support undergraduate teaching), while other activities (e.g., secondary outreach) tended to be reduced or eliminated. The TIR can serve a critical role as a physical teaching ambassador who explicitly encourages undergraduates who enjoy early teaching experiences (like being a LA) to consider a career in teaching. Without someone in this role, it may be hard for students to make this connection themselves (for example, at Towson and MidTenn whose TIR was slightly less accessible with a location in education).

Sites can strategically leverage this affect by consciously tying “new” activities to essential departmental tasks. For example, both VTech and BU started Studio Physics and an LA program around the same time as PhysTEC. For both institutions, the introductory course transformation helped increase student engagement and interest in physics in general—cutting failure rates and increasing the number of majors. Attracting more students into physics creates a bigger pool of potential teachers; however, the number of teachers does not automatically increase linearly with the number of majors. For example, undergraduates may enjoy serving as LAs but without explicit encouragement, they may not associate that with a career in high school teaching.

While introductory course transformation is not *sufficient* to increase the number of potential teachers, it can be leveraged for sustainability purposes. Delivering high-quality undergraduate courses falls directly within a department’s mission, unlike producing secondary teachers. If the TIR’s expertise can be utilized to lead these efforts, it can cause an increased reliance on their expertise. Specifically, the TIR at VTech had a big role in starting studio and the LA program. She recruited undergraduates through multiple channels and taught courses in physics, physics pedagogy, first year experience, and physics outreach (designed to improve communication skills regardless of career path). She advised the Society of Physics students and hosted pizza parties where undergraduates can learn about outreach opportunities. She had a highly visible role in the department, impacted essential departmental activities, and VTech made her a full time instructor before matched funding ended.

In contrast, the contributions of the BU TIR could be considered “auxiliary” to typical departmental duties. He did provide advice for starting studio and provided mentoring for the LAs, but he spent most of his time recruiting career changers into the Masters of Teaching program and developing a network of over 200 local teachers. While these accomplishments are impressive, a department head at a research institution may not find recruiting future teachers vital to university operation. The department did not offer to make his role permanent. When PhysTEC funding ran out, the continuation of his position was dependent on finding external funding.

Both VTech and BU were successful in producing over five physics teachers a year, but because the VTech TIR’s activities were tightly tied to essential, undergraduate activities and eliminated the potential obstacle of her being auxiliary rather than essential, it was easier for VTech to justify integrating her as a full-time instructor in the physics department.

### Institutional commitment

A strategic initiative that enhances the established organizational culture has a high probability of success. When the change conflicts with the organizational culture, culture can diminish the power of the change initiative (Mento et al. 2002). Our study found that departmental culture resisted significant changes during the grant. UMN and BU’s PhysTEC efforts and sustainability continued to be threatened by negative attitudes toward teaching as a profession. The three most successful sites had PIs of PhysTEC serving as department heads at some point during the grant, where they made sure teaching was seen as a valuable potential career path.

UMN and BU achieved success during the grant period due to extremely motivated individuals who applied for external grants and recruited career changers into teaching. However, the institutional culture limited PhysTEC’s impact on undergraduates and threatens ongoing sustainability. The notion that undergraduates do not attend these institutions to become teachers stubbornly persisted. At BU, recruiting efforts failed to gain widespread acceptance among undergraduates because of the high tuition, additional time required for certification, and bias against physics teaching as a career. Furthermore, PhysTEC efforts remained relatively isolated to a few key individuals who were already invested in education. Similarly, at UMN, the TIR says there is some continuation of PhysTEC efforts but.

we’ve had a really hard time finding a lot of people that want to become high school physics teachers at the U. Kids that want to go into high school teaching tend to go to smaller colleges where you can get a license after your bachelor’s degree instead of having to get a master’s like you do at the U. That part of it has been difficult, you know recruiting kids that want to be in high school teaching that’s just not why kids go to the U.

While BU and UMN had some success during the grant because of dedicated efforts of key players, both programs are at a high risk for discontinuation if key people leave or without external funding to support activities.

### Status and support of faculty champion

PhysTEC succeeded when the faculty champion had enough authority, clout, and/or respect from a research track record (like CSULB, VTech, UMN, and MidTenn) to highlight physics teacher preparation as a valued departmental activity. Not coincidentally, the three most successful sites (MidTenn, CSULB, VTech) had PhysTEC PIs that served as department chairs at some point during the grant. As departmental leadership, PIs have the influence and funding to help change departmental norms to value physics teacher preparation. For example, the current Associate Chair of Physics at VTech states,

I think we’re gradually getting most faculty on board with the way that these things should be done, and the current chair of the department certainly likes our teaching and so, I think the atmosphere and the dynamic within the department is to continue these things. Exactly who will do them is not exactly obvious… I’m not exactly sure how it would work out, but I think that the atmosphere and philosophy of the department has gone beyond some barrier, which I think could sustain this even in our absence.

None of the sites that struggled with sustainability had a PhysTEC champion in a leadership position, and the champion might have lacked credibility due to disciplinary research. At BU, the champion in physics was a Master Lecturer. At Towson, the co-PIs were science educators, one of whom was a new employee. At UMN, the champion was research faculty in Astrophysics but did not have a leadership role. Seymour (2001) shows that the research credentials of the champion are more effective in persuading other faculty about the worth of a teaching innovation than either the data that supports its efficacy or video that demonstrates its merits or shows that students like it.

### A unified vision and clear communication

Successful sites had clear communication to ensure a unified vision (Chasteen et al. 2018) between physics, education, and administrative stakeholders (which include the UTeach initiative for two sites). A team approach to change helps ensure sustainability, but a larger collaboration can complicate creating a common vision (for example, with UTeach). At VTech, the initial PI for PhysTEC had shifted to another university but there was someone in physics who could step up and maintain momentum. VTech also benefitted because this person had a long-standing friendship with a colleague in education leading to a tight collaboration departments. At CSULB, the physics department head knew that her experience with educational initiatives was limited and worked closely with a colleague in education to outline realistic, achievable goals.

Towson’s historic reputation as a teaching college and the long-standing tradition of having science educators within the department seems like fertile ground for sustained change, but it was not enough to compensate for communication challenges and misaligned goals between PhysTEC and UTeach. This contrasts with MidTenn when the two efforts complemented each other from the start.

At MidTenn, the start of PhysTEC overlapped with the start of UTeach so there was a high degree of collaboration from the beginning. The physics department used the UTeach Master Teacher to adopt the TIR role in the physics department. While his office location in the College of Education makes him slightly less accessible, the Master Teacher still could provide discipline-specific coaching and mentoring. The UTeach co-director actively recruits students into physics teaching, describing favorable career prospects and recommending physics as a major. Similarly, the PhysTEC champion and physics Chair make announcements in introductory physics courses encouraging students to consider taking courses in education. While the physics Chair is not technically UTeach leadership, he attends monthly meetings and has a strong relationship with the UTeach director to ensure continuity and synergy between programs. Having multiple persons communicate the message to various stakeholders likely contribute to MidTenn’s success.

In contrast, at Towson, PhysTEC preceded UTeach and structural differences made communication difficult between parties. Specifically, Towson houses science education specialists within the departmental discipline. This worked well for PhysTEC where efforts targeted the physics department but UTeach was based in the education department on campus which limited interaction with the science education specialists in the physics department. UTeach offers a standard set of courses through the education department. In the interviews, the PhysTEC PIs described feeling like the president implemented a “franchise” structure, without asking for input from themselves. One of the PIs explained, UTeach has to.

take care of very specific assessment things. So they didn’t necessarily have time to integrate or think about what we had done before. It was just really unfortunate that we had spent three years developing expertise on physics education and then it just disappeared as soon as UTeach started.

While the physics education specialists tried to attend some UTeach meetings, it did not lead to productive collaborations. UTeach prepares STEM teachers in general and our Towson interviewees did not believe UTeach prioritized recruiting students into high needs areas like physics or chemistry. They lost their TIR when PhysTEC funding ran out, workshops for mentor teachers stopped, outreach decreased, and the pedagogy course has also faded over time. While Towson does have a thriving LA program funded by the university, without a TIR, students do not tend to connect being an LA with pursuing physics teaching as a career.

Situational and structural differences between Towson and MidTenn contribute to the results but these examples demonstrate the importance of meaningful collaboration and aligned vision when trying to create synergistic connections between two funding initiatives.

## Limitations

Studying educational reform attempts is inherently difficult, due to their complexity and highly contextual nature. History, culture, interactions, internal and external events are an integral part of the change literature and cannot be ignored. No amount of empirical research will lead to a simple recipe for a fool-proof change strategy that works at all institutions. We are not suggesting that sustainability can be achieved by exactly replicating what worked elsewhere or by following a simple sequential process. Instead, we wanted to communicate that change is a process that starts by analyzing whether the climate will support the change.

Throughout the paper, we included insights from a variety of change literature under the belief that the field needs to consider reforms from multiple frameworks that were developed in diverse contexts. As Sidorko (2008) points out,

Perhaps the key to successfully implementing change models lies not so much in following them prescriptively (although many of their authors advise this as the best method), or as a panacea, but in the ability to implement them selectively and adaptively in order to best match the culture and environment of the organization (p. 316).

The case studies and change model presented here are included so reformers increase their awareness of possible ways to cultivate fertile ground for sustainable change from the start.

While our data was acquired over time and we have tried to ensure that our data reflects multiple perspectives, these case studies reflect a small number of people and events. While we spoke to multiple people at each institution, the perspectives reflected here are still limited.

## Conclusion

A central claim of this paper is that sustainable change requires a climate that aligns with the reform. Successful sites realized PhysTEC could be a solution to institutional needs, such as producing more majors, increasing the quality of undergraduate education, and providing more career options for graduates. This climate includes both values and culture. Values are explicit or implicit expressions of the “desirable” that influence individuals’ means and ends of action (Kluckhohn 1951; Berson et al. 2008). Values affect how events are interpreted and can influence the amount and type of information that people process. Culture represents the shared perceptions and orientations toward meaning within the specific domain of departmental activities, which tend to be relatively dynamic and sensitive to external influences (Berson et al. 2008). When champions individually valued teacher preparation were in leadership positions, they could create a shared sense of meaning around how these activities could enhance existing activities and set up structures to embed these values within the departmental culture.

If the organization or champions in leadership roles do not value teacher preparation, grant funding will likely not change that. While BU and UMN successfully recruited career changers for teaching certification, the attitude that students do not attend those universities to be teachers persisted, and led to precarious sustainability. Having a well-respected faculty champion in a strategic position who supports teacher education can help increase visibility of the cause and set up structures to further the cause (for example, with hiring and promotion). Reform activities that simultaneously benefit undergraduate education and physics teacher preparation are easier to sustain, partially because of alignment around a unified vision that encompasses the reform.

The PhysTEC grant provided resources to sites that were primed to flourish. The grant brought financial resources, prestige, and support to bring physics teacher preparation to the foreground. The selection criteria funds sites already have some ingredients for success: a departmental champion, support of the physics department head, a collaboration with the School of Education, and a commitment from higher administration in the form of matched funding. PhysTEC supports sites in defining their vision, providing advice and connecting sites in a supportive community.

A common vision and collaboration is important for widespread buy-in. The weakest point for sustainability of these programs seems to be attracting new champions to the cause, so a “team effort” to reform is advantageous (Chasteen et al. 2018). While a larger team may make it harder to come up with a unified vision, it can protect against faculty and administrative turnover and involve more widespread buy in. Interviewees from MidTenn, VTech, and CSULB all said that PhysTEC has become engrained in the departmental culture but interviewees could not name who would undertake the PhysTEC activities in their absence. Beyond that, sustaining teacher preparation programs is possible and the presence of all of these elements help.

Interestingly, the sustainability in our study (with three sites institutionalizing changes, two maintaining teacher production for the moment and one discontinued) does not appear to be a significant improvement over what was reported by Scherr et al. (2014). In Scherr’s et al. (2014) study, out of eight sites, four further increased teacher production, three maintained an increase and activities at one institution were largely discontinued. While we cannot claim statistically significant changes in sustainability under the new funding structure because of small numbers in this qualitative study, it seems the new requirement for a sustainability plan does not necessarily change the potential for sustainability that existed before the requirement. The matched funding requirement does ensure commitment from higher administration, increasing the likelihood that physics teacher preparation is valued by the institution. However, it seems more important to assess the general attitude students and faculty members have toward teaching as a profession. The PhysTEC team should also comprise of people with enough position power to promote this cause within the departmental value system, enough expertise to make informed decisions about what activities to invest in, and enough credibility and leadership to inspire progress (Kotter 1996).

Although this study examined the PhysTEC reform, we believe results may apply to sustaining other reforms that hope to advance undervalued activities at universities. A key message is change agents should focus on seeding reform efforts at sites where further growth and propagation is likely. The change must align with the existing departmental and institutional culture and a well-respected champion can help further advance the change. Activities should be undertaken strategically to support existing departmental activities as well as furthering the reform effort.

## Abbreviations

AAPT: American Association of Physics Teachers
LA: Learning Assistant
NSF: National Science Foundation
PhysTEC: Physics Teacher Education Coalition
TIR: Teacher In Residence
